# Challenges to the real-world delivery of brief alcohol interventions in the custody suite: qualitative study

**DOI:** 10.1192/bjb.2024.48

**Published:** 2024-10

**Authors:** Manuela Jarrett, Thomas Mills, Jaimee Mallion, Susie Sykes, Jane Wills, Eddie Chaplin

**Affiliations:** 1 University of Birmingham, Birmingham, UK; 2 London South Bank University, London, UK; 3 National Children's Bureau, London, UK

**Keywords:** Police custody, alcohol, brief intervention, public health, qualitative

## Abstract

**Aims and method:**

The aim was to evaluate an innovative pathway in police custody suites that aimed to specifically address alcohol-related health needs through screening and brief interventions by police custody staff. This paper presents a qualitative investigation of challenges involved in implementing the pathway. Qualitative interviews were carried out with 22 staff involved with commissioning and delivering the pathway; thematic analysis of interview data was then undertaken.

**Results:**

An overarching theme highlights the challenges and uncertainties of delivering brief alcohol interventions in the custody suite. These include challenges related to the setting, the confidence and competence of the staff, identifying for whom a brief intervention would be of benefit and the nature of the brief intervention.

**Clinical implications:**

Our findings show that there is a lack of clarity over how alcohol-related offending can be identified in police custody, whose role it is to do that and how to intervene.

A recent independent review of UK national drug policy by Dame Carol Black commissioned by the government stated that services for drug and alcohol users were ‘not fit for purpose’^1^ and led to new investment and ambitious targets for diverting people from the criminal justice system into treatment.^2^ Alcohol is the most prevalently used substance, with evidence showing that between 64 and 88% of adults in police custody settings meet criteria for risk drinking^3^ and alcohol-related crime was costed at £11 billion per year in 2010–2011.^4^ It is implicated in over half of all violent crimes,^5,6^ particularly domestic violence^7^ and sexual offending.^8^ There is also evidence that combined use of alcohol with other drugs can exacerbate the risk of offending/violent behaviours.^5,9^

The past few decades have begun to see a shift in the perception of alcohol as a problem of individual addiction to its recognition as a public health concern that is associated with a range of societal harms.^10–12^ The focus is now on early intervention and prevention of harm,^12^ including the use of brief interventions.^13^ Brief interventions involve asking screening questions to determine whether alcohol consumption exceeds low-risk limits, and if so, structured advice and counselling are offered^14^ and can lead to referral to specialist services.^15^ They aim to mitigate behaviour in individuals drinking above low-risk levels by providing them with tailored information in a personalised and non-judgemental fashion.^16^

In late 2021, a National Health Service (NHS) public health commissioning body in a large non-metropolitan area in England, along with the local Office of the Police and Crime Commissioner (OPCC), commissioned an innovative pathway in police custody aimed at reducing reoffending by taking a targeted and layered approach to individuals with substance use problems, including alcohol. Previously the local service had been a continuation of the Drug Intervention Programme (DIP), initially set up to mandate treatment for opiate and crack cocaine users arrested for acquisitive crimes.^17^ However, concerns over low footfall, inefficient use of staff resources and the disruption caused by the COVID-19 pandemic led to the development of the new pathway. An additional aim was to include a focus on individuals with alcohol, not just drug, problems. The aim was for police custody suite staff to screen detainees who presented with evidence of alcohol use and to triage them to three levels of intervention: universal (brief intervention for all detainees presenting with evidence of alcohol use), delivered by custody staff; tailored (support into treatment for those who voluntarily wish to enter it); and intensive (aimed at prolific offenders, with referral to a multi-agency panel, mandatory assessment and assertive outreach).

The Public Health Intervention Responsive Studies Team (PHIRST) at London South Bank University led an evaluation that was co-produced with the local public health commissioning body and other local stakeholders to evaluate the new pathway. However, the pathway was not implemented fully owing to various challenges, which included high staff turnover among both the custody suite and the local substance misuse services. This paper presents a process evaluation of the contextual factors that inhibited effective implementation of the alcohol component of this intervention. Reflection on the alcohol harms component of the pathway may provide insight into some of the challenges to addressing alcohol-related health needs in custody.

Ethical approval was secured from the School of Health and Social Care Ethics Panel at London South Bank University (ETH2122-0131 and ETH2122-0176). This study was carried out at PHIRST South Bank, based at London South Bank University.

## Method

### Approach

A logic model of the pathway concept and anticipated mechanisms and outcomes was produced during three co-production workshops. These involved police, clinical and public health staff as well as people who use the service (details available from the corresponding author, M.J.). The logic model articulated the theory of change – that alcohol harms could be reduced by targeted interventions in the custody suite and by police and health services working together. The evaluation approach thus focused on understanding the experiences of people working within and using the service and the interview schedule explored perceived roles and skills and the processes of the pathway. Evidence of the value of logic models as guiding frameworks for implementation and evaluation of public health programmes is already growing in the literature.^18–20^

### Participants and recruitment

Recruitment was organised by local partners at a police custody suite and proceeded via purposive sampling to ensure that key stakeholder agencies (public health commissioning, local substance misuse service, OPCC and the police) and staff groups who work in police custody (police, local substance misuse service staff and NHS Liaison and Diversion services) were included. All participants provided written informed consent. Despite regular liaison with keyworkers, there were several challenges to recruitment of people using the service: for example, contacting those individuals proved very difficult and one-to-one interviews were not permitted either onsite or elsewhere for safety reasons. Interviews were carried out with senior as well as front-line staff who were involved in developing or implementing the proposed pathway. Most interviews took place digitally via Microsoft Teams, with just one exception, which was a telephone interview.

### Interview protocol

A semi-structured interview schedule, which was informed by the logic model, was developed by the research team to explore the pathway's implementation and how it was perceived. The interview included questions pertaining to: professional role congruence; skills and skill gaps; processes for identifying and managing alcohol users in the custody suite; and feasibility of brief interventions for alcohol use. Interviews were recorded and transcribed for analysis.

### Data analysis

Members of the research team familiarised themselves with the data and developed a coding framework through consensus discussion. This was flexibly applied to all data using NVivo 12 and adapted to capture categories of interest. All alcohol-related references in the interviews were extracted and then underwent further analysis and coding. Familiarisation with these extracts was followed by open coding to identify initial broad categories of themes. These categories then underwent reiterative coding as patterns in the data were identified in relation to the categories, which were then conceptualised as themes. The themes were revised and finalised through further review and discussion within the team.

### Reflexivity

The research team comprised individuals with varying professional backgrounds (public health research, psychology, mental health nursing and criminal justice research), which helped the team to maintain a reflexive approach to the development of the interview schedule, analysis and writing up of the study. Existing assumptions were challenged via the different perspectives within the team. The interviews were carried out by three members of the team (T.M., J.M. and M.J.) and all members of the team contributed to the theme development.

### Findings

Twenty-two participants were interviewed. Participants comprised three public health commissioners, one OPCC commissioner, seven police officers, seven staff from the local substance misuse service and four NHS Liaison and Diversion staff. We were unable to interview people using the service, owing to the recruitment challenges outlined above. The following labels are used to represent the participants: LGPH (local government public health), PS (police staff), SW (substance use worker) and L&D (NHS L&D). Three commissioners were interviewed twice at different stages to gain insight into their perception of implementation challenges and whether and how they might respond in their commissioning arrangements. In total, 25 interviews were carried out, ranging from 23 min to 1 h 10 min.

### Challenges in brief alcohol intervention delivery

The overarching theme identified in the data was challenges in delivery of the brief alcohol intervention. This theme included four sub-themes: (a) where to intervene: advantages and disadvantages of the custody suite as the point of intervention; and (b) who to target: identifying people in police custody who may benefit from a brief alcohol intervention; which had an overlap with (c) how to intervene: advantages and disadvantages of layered interventions and (d) who should intervene: staff roles in intervention delivery.

#### Where to intervene: advantages and disadvantages of the custody suite as the point of intervention

Questions were raised as to whether the custody suite was the most appropriate place to intervene for people with alcohol-related health needs whose drinking was risky but not at the threshold of alcohol dependency. Indeed, whereas the identification and management of people with alcohol dependency was established in the custody suite, there were many practical challenges to addressing this other, larger group. Challenges included people being too intoxicated to engage with any intervention and, when they are more recovered, being keen to leave the station rather than remain and discuss their alcohol use. A participant commented:

‘There's a practical problem with alcohol though compared to drugs in that they're probably inebriated at the point at which they're in the custody suite, so what can you actually do with them anyway?’ (LGPH2b).

However, it was also acknowledged that interventions at a crisis point in a person's life can provide the opportunity for powerful moments of reflection. Not intervening in the custody suite could therefore be a missed opportunity to engage with the individual, as a participant observed:

‘Something has to happen for them to stop, take stock and reflect on that and say this is time for me to do something’ (SW3).

An additional point that participants disagreed on was whether there was enough of a need in the custody suite to warrant specialist resource input. Some were concerned about the high level of need:

‘The referrals in regard to alcohol is a lot less, so that's the issue. We know the people and there are a hell of a lot of people that are going through the custody suites that we don't know that are on alcohol and those are the differences really’ (SW3).

But not everyone agreed:

‘The quality of the data isn't robust enough to ascertain needs around alcohol, numbers being arrested around alcohol. It is that the police want somebody there in custody, but actually the arrest data also doesn't warrant people being there’ (LGPH1b).

How many people passing through the custody suite present with alcohol use is a crucial issue since it dictates the level of resources required to address the need. One of the challenges in delivering brief interventions for alcohol in this setting was to identify who needed the interventions.

#### Who to target – identifying people in police custody who may benefit from a brief alcohol intervention

The new pathway took an innovative approach in that it specifically included individuals presenting with alcohol use, rather than focusing mainly on those with drug use; and it took a layered approach to addressing the individual's needs, according to the impact the alcohol was having on their offending behaviour. There were three levels of intervention, ranging from (1) psychoeducation and brief intervention, to (2) support for those seeking help, to (3) a multi-agency panel and mandatory initial assessment followed by assertive outreach support for a limited period by a specialist worker. Custody staff were most confident with identifying those requiring the level 3 intervention, which was aimed at individuals being repeatedly arrested for crimes committed while under the influence of alcohol. However, for levels 1 and 2, identification was more challenging.

At the lowest level, level 1, the aim was to provide basic psychoeducation (information about alcohol and its health impact) to everyone with alcohol-related health needs. This would include individuals held in police custody and later released without charge. This contrasted with the previous way of working in that ‘nothing would be happening with people that were drinking’ (SW1). When asked about how detainees who had used alcohol were identified, we were informed that custody staff were required to ask some ‘basic questions around drugs and alcohol’ (SW1) and that these were part of the standard police risk assessment. Police participants described confidently being alert to detainees presenting with harmful drinking levels that were a threat to health. One officer told us:

‘So somebody who is an alcoholic we'll manage very closely, they will speak to the healthcare practitioner and they will be monitored very closely to make sure that they don't withdraw because we realise the dangers there are within alcohol’ (PS6).

However, it was unclear how, and even if, alcohol use that exceeds the low-risk threshold was identified. Further, participants were unlikely to broach the topic with the detainee if they believed that the alcohol-related offence was an isolated incident (e.g. ‘your drink-drivers that come in and say it's a one off’ (L&D2)). Alcohol was viewed as different from other substances in that it is legal, easily accessible and the boundaries between recreational and dependent or problematic use are blurred. The expectation was that detainees could request help if they felt they needed it. One participant commented:

‘Unless they say that they regularly consume alcohol or take substances, or, like you say, aren't repeat offenders, then I'm sure there are people who probably would go under the radar and would be missed’ (L&D1).

Level 2 intervention was important as it could potentially detect those who had not been identified previously whose offending may be related to alcohol use. A participant explained:

‘Level 2 is people who have been arrested, gone into custody, they've met their trigger offence criteria, they get drug tested and they are positive for a drug, let's say cocaine for instance [ … ]. The difference in our new model was we also wanted to target alcohol service users as well’ (SW1).

‘We were able to see some people who were coming for alcohol-related crimes or offences that they had committed which historically we had never seen before’ (SW1).

This level of intervention involved supporting offenders to voluntarily access treatment, and giving them access to support groups and a volunteer to help them along the way. However, although participants were able to describe clear ‘trigger offences’ that would result in referrals to drug services (e.g. a robbery to acquire money to buy drugs), this was not the case for alcohol. Even offences directly related to alcohol, such as drink-driving, were not considered a trigger offence. It was unclear, for example, whether an individual detained for a domestic violence offence while under the influence of alcohol would trigger a referral to services. One participant commented:

‘I'd expect most of late-night weekend custody suite attendances to be alcohol related, if I'm totally honest I can imagine that's the case, so to not see the figures coming through I find that quite odd, so my gut reaction is they're being missed’ (LGPH2b).

Participants in the local substance misuse services were confident that those who met level 3 criteria were likely to be identified, but that level 2 people could potentially be missed:

‘So, level 2 [ … ] it will be dependent on people who continue to go through the system and we need to have recorded that they need to have gone through the system, which could be a risk because if custody sergeants don't record them coming through for an alcohol need then we'll never pick them up and they could have repeatedly come through and they have got an alcohol need’ (SW1).

Some participants reiterated the view that problematic alcohol use was being missed:

‘I can imagine alcohol being a factor in a huge amount of arrests and offences. In fact, I've got in my head, I saw somewhere – it was in the 70s or 80%. So alcohol's always been missed’ (LGPH1b).

The layer of intervention was determined by the detainee's history of offending in relation to their alcohol use. This also affected the type of intervention – whether information provision alone or a more prolonged conversation about alcohol use with a substance misuse worker was needed. The types of intervention, as discussed in the next theme, were a source of contention among participants.

#### How to intervene: information provision – ‘not ideal but certainly more reasonable’

The layered intervention approach called for escalating levels of input depending on the history of the detainee and how well-known they were to police for alcohol-related crimes. At a basic level, all detainees who responded affirmatively to questions about alcohol use would be provided with information about alcohol in the form of a leaflet or information on a digital tablet. This was intended to help increase the individual's awareness of how alcohol may be affecting their behaviour. As one participant told us:

‘It may well be a telephone number, it may well be a leaflet, it may well be a conversation, it may well be the fact that someone has injured themselves – they've fallen over and this is the fifth time they've done it and it was particularly dangerous this time because they bumped their head or they were in a fight. Sometimes you can put the dots together and people go “actually this is all about your alcohol use”’ (SW3).

Information provided at level 1 could then potentially lead to a referral to the local substance misuse service:

‘If any of that information has resonated with that person, we were anticipating that actually that person might say yes, I want to go to [local substance misuse service] and it would have been simple to take their name and contact details and we'll do all the rest. So, it is a very universal service at level 1’ (SW1).

However, participants commented that leaflets and digital information rely on the detainee engaging with the material given:

‘It will work for some; it won't work for others, is the truth, I think. I would say, whilst they're in Custody, they're provided with a leaflet to take: I have to think, “Yes”, it does [work] because, actually, they can take it home and, also, somebody within that household or residency might pick it up and see it, provide support, [...] maybe relationship support to the individual and support them to seek further help. So, yes. Others will just bin it or ignore it or might not even take it’ (PS2).

There are also other challenges with just providing written information to the detainee to read, as one participant noted:

‘I think in an ideal world it is not just information that we want to give people because we don't know people's reading age. I think the average adult reading age in the UK is five or six, I believe, years old so there are people who are potentially going to be reading information who may not even know how to read, so we are not saying that this information is going to be read by everyone and they are going to get it’ (SW1).

This participant acknowledged that, although it would be preferable for a member of staff to go through the information with the detainee, this would require more staff resources and was simply not feasible. So, although a face-to-face conversation might be preferable, provision of information in paper or digital form was viewed as a ‘second-best option. It's not an ideal option, nowhere near that but it certainly is more reasonable’ (SW1).

However, police participants perceived face-to-face conversations about alcohol use as requiring a specialist skill set acquired through the relevant training, which they did not have. This then raised the question of who should be delivering the interventions in the custody suite.

#### Who should intervene – staff roles in intervention delivery

The new pathway included the expectation that custody officers would be able to identify individuals who had used alcohol and deliver a brief intervention to them. This was seen to be straightforward: ‘all we wanted the sergeants to do is ask them the basic questions around drugs and alcohol’ (SW1), which was perceived to be part of the usual risk assessment and routine questions asked by custody staff.

Since referrals to the substance misuse team came via the police based on how detainees had responded to questions, the delivery of a brief intervention in the form of information via leaflets or tablets was therefore not seen by the public health commissioners of the intervention as particularly onerous. A participant told us:

‘What they have historically been doing for a number of years is asking one or two questions – have you got a drug issue or a drug and alcohol issue? So, we weren't really moving the goalposts from that’ (SW1).

However, police participants thought this role should fall to healthcare workers. One officer commented:

‘You are asking about drugs and alcohol; you're asking about something that a health professional potentially needs to do’ (SW1).

‘So I think it is beneficial to have that softer skill set as well, of saying let's help out, they are available for you if you want to engage, and that goes a long way as well’ (PS3).

Participants added that such a conversation would require some level of skill and therefore training. One participant said that the idea of police delivering brief interventions ‘looked great on paper but, yes, there's wider issues there in terms of implementing it’ (LGPH3). There was a sense that an environment conducive to supporting detainees would be facilitated by police having a better understanding of how to identify issues and what advice to provide to the person:

‘If people feel comfortable and competent to have those conversations, everyone can be trained to have a conversation around drugs and alcohol but you have got to feel comfortable and know how to have that conversation. That's not to say that in future the detention officers won't be able to have one but there's got to be the opportunity to train them up to have that conversation’ (SW1).

Further, police participants commented that, as people in authority, they were perhaps not the right people trained to deliver interventions:

‘The uniform in some respects is quite a barrier [ … ] whereas the drug and alcohol worker is much more able to [ … ] be the independent ear’ (PS3).

## Discussion

This paper presents findings pertaining to the challenges that inhibited the effective delivery of a pathway for addressing alcohol-related health need in the custody suite. This new service model required police custody staff to identify detainees whose drinking was above low-risk thresholds but not at the level of alcohol dependency, to enact decisions regarding triage of interventions as well as deliver brief interventions. There was consensus in relation to the difficulties of delivering such a pathway in a police custody setting.

Although public health commissioners thought it would be possible initially, all agencies queried whether police custody was the best place to deliver brief alcohol interventions. Participants described detainees as not being amenable to conversations about their drinking, however brief, as they are either too intoxicated or, once recovered, too keen to leave. These findings echo those of other research on brief alcohol intervention delivery in the custody suite.^21,22^ In addition, although detainees who are too inebriated to hold a conversation would presumably be easy to identify, it was unclear how those with less obvious presentations, but still above low-risk thresholds, would be identified.

The expectation was that police custody staff would be able to triage detainees according to the pathway level required and also deliver brief alcohol interventions to all. Public health commissioners of the pathway argued that police custody staff already asked basic questions about alcohol and substance use, so this was within their remit. However, research shows that this task of opening a conversation about alcohol use with a view to encouraging self-reflection and exploring changes in behaviour needs counselling micro-skills that require motivation, training and experience to build competency.^23,24^ It is unlikely that police custody staff would be capable of delivering brief alcohol interventions effectively, therefore, without extensive training and a fundamental shift in their role towards public health. One suggestion was to provide detainees with leaflets that they can take away. However, some participants noted that this approach lacked a necessary proactive element to engage with detainees, who can simply discard the information. McGill et al^25^ have argued that psychoeducation about alcohol needs to engage the person receiving the information and should be supported by a discussion, to facilitate both teaching and learning, because ‘information delivered does not equate to information received’. In addition, one participant noted that detainees often have low reading levels. Although there are no literacy data on those in police custody, the Ministry of Justice reported that 61% of a large sample of adult prisoners in England in 2021–2022 had a reading age lower than that of 11-year-olds.^26^ Health literacy rates among this population are likely to be even lower.^27^ This does therefore have implications for delivering brief interventions as written information. Further, if staff do not have the skills to have the conversation, it is likely that they underestimate the numbers of detainees with alcohol-related health needs.

McCambridge^16^ argues that although individuals are willing to be asked questions about their alcohol use, acknowledging that their use may exceed any risk threshold and that an intervention may be required is a different issue altogether. The second level of the pathway involved precisely this – offering further support that detainees could voluntarily take up if they wished. The stigma associated with alcohol dependency means that people are unlikely to acknowledge a need for support with reducing their alcohol use when they do not meet the highest-risk thresholds. This will likely serve as a barrier to entry at level 2. Further, triaging to the second level of the pathway was linked to this being the detainee's first or second offence linked to alcohol use. However, the first arrest does not necessarily indicate it is the first time the offence has been committed. For example, approximately 19% of drink-drivers admit to driving over the legal limit before their first arrest,^28^ and high-risk victims of domestic abuse live with the abuse for an average of 2.3 years before reporting it to the police.^29^ Although certain offences triggered referrals to services for drug use, there were no identified offences that would trigger a referral to services for alcohol use even if obviously related (e.g. drink-driving). Arguably, it may be helpful to consider the possibility of referral at the first detention, as it is unlikely that this is the first time the offence has been committed and if the offence is related to alcohol, this is potentially a window of opportunity to intervene to prevent further offences.

Front-line approaches to managing alcohol-related crime are affected by commissioning and budgets determined by national policy. Since publication of the last UK Government Alcohol Strategy over a decade ago^30^ there has been little in the way of initiatives to address alcohol consumption in the UK. This is despite alcohol being strongly linked to crime.^5,31^ In February 2023, the government announced a £421 million investment to boost drug and alcohol treatment and recovery services.^32^ The barriers highlighted by participants in this study, including the motivation and skills of staff, are reminiscent of those found by Best et al two decades ago,^33^ and show that little has changed in terms of the barriers in this setting. Alcohol and crime are closely linked, and it can be argued that acknowledging and attempting to address alcohol use in a custody setting is a valuable opportunity that should not be missed.

### Strengths and limitations

The evaluation included interviews with stakeholders from across staff fields and at all levels, from front-line to senior management and commissioners. However, we were unable to interview those using the service (detainees), whose views would have offered valuable insights into the difficulties of implementing this intervention. The difficulties we faced in recruiting alcohol detainees, such as making contact following release from custody, reflect some of the identified barriers in the pathway. A further limitation is selection bias, since all front-line staff who took part were approached by email and via snowballing from other participants. There may be inherent differences between those who agreed to take part and those who did not.

### Implications and recommendations

This evaluation has broad relevance to public health commissioning, the Office of the Police and Crime Commissioner (OPCC), substance misuse services and the police force, given the heightened interest in criminal justice pathways into treatment following Dame Carol Black's report focused on dependent users of alcohol and individuals ‘struggling with alcohol’^2^ (p. 31), which many people who have harmful alcohol use may not relate to. The evaluation has highlighted some important knowledge gaps. Delivering brief interventions requires skill and therefore training^34^ but there is little understanding of how what McCambridge^16^ calls the ‘the causal chain’ works. This refers to various and probably nuanced stages between training a member of staff to deliver a brief intervention and that intervention reducing a person's alcohol consumption. The original idea underpinning the pathway that police custody staff would have a role in delivery of a brief alcohol intervention requires further consideration as we found little to suggest that they were either able or willing to fulfil such a role.

## Data Availability

Data are available on reasonable request.

## About the authors

**Manuela Jarrett** is Assistant Professor in Mental Health Nursing in the Institute of Clinical Sciences, College of Medical and Dental Science, University of Birmingham, Birmingham, UK. **Thomas Mills** is a senior research fellow in the Public Health Intervention Responsive Studies Team, London South Bank University, London, UK. **Jaimee Mallion** is a senior research and development manager with the National Children's Bureau and an honorary research fellow in the Department of Psychology, London South Bank University, London, UK. **Susie Sykes** is Professor of Public Health and Health Promotion in the Public Health Intervention Responsive Studies Team, London South Bank University, London, UK. **Jane Wills** is Professor of Public Health Promotion in the Public Health Intervention Responsive Studies Team, London South Bank University, London, UK. **Eddie Chaplin** is Professor of Mental Health in Neurodevelopmental Conditions in the Institute of Health and Social Care, London South Bank University, London, UK.
